# 3D printing as a pedagogical tool for teaching normal human anatomy: a systematic review

**DOI:** 10.1186/s12909-023-04744-w

**Published:** 2023-10-20

**Authors:** Eléonore Brumpt, Eugénie Bertin, Laurent Tatu, Aurélien Louvrier

**Affiliations:** 1 University of Franche-Comté, 19 rue Ambroise Paré, Besançon, 25000 France; 2 Radiologie, CHU de Besançon, Besançon, 25000 France; 3 Laboratoire Nano Médecine, Imagerie, Thérapeutique, EA 4662, University of Franche-Comté, 16 Route de Gray, Besançon, F-25000 France; 4 Anatomy Department, UFR Santé, 19 Rue Ambroise Paré, CS 71806, Besançon, F25030 France; 5 Chirurgie Maxillo-Faciale, Stomatologie Et Odontologie Hospitalière, CHU de Besançon, Besançon, 25000 France; 6 Neurologie, CHU de Besançon, Besançon, 25000 France; 7 Laboratoire de Neurosciences Intégratives Et Cliniques, University Franche-Comté, EA 481, Besançon, F-25000 France; 8 Plateforme I3DM (Impression 3D Médicale), CHU Besançon, Besançon, 25000 France

**Keywords:** Anatomy, Learning, Review, Teaching, 3D printing

## Abstract

**Background:**

Three-dimensional-printed anatomical models (3DPAMs) appear to be a relevant tool due to their educational value and their feasibility. The objectives of this review were to describe and analyse the methods utilised for creating 3DPAMs used in teaching human anatomy and for evaluating its pedagogical contribution.

**Methods:**

An electronic search was conducted on PubMed using the following terms: education, school, learning, teaching, learn, teach, educational, three-dimensional, 3D, 3-dimensional, printing, printed, print, anatomy, anatomical, anatomically, and anatomic. Data retrieved included study characteristics, model design, morphological evaluation, educational performance, advantages, and disadvantages.

**Results:**

Of the 68 articles selected, the cephalic region was the most studied (33 articles); 51 articles mentioned bone printing. In 47 articles, the 3DPAM was designed from CT scans. Five printing processes were listed. Plastic and its derivatives were used in 48 studies. The cost per design ranged from 1.25 USD to 2800 USD. Thirty-seven studies compared 3DPAM to a reference model. Thirty-three articles investigated educational performance. The main advantages were visual and haptic qualities, effectiveness for teaching, reproducibility, customizability and manipulability, time savings, integration of functional anatomy, better mental rotation ability, knowledge retention, and educator/student satisfaction. The main disadvantages were related to the design: consistency, lack of detail or transparency, overly bright colours, long printing time, and high cost.

**Conclusion:**

This systematic review demonstrates that 3DPAMs are feasible at a low cost and effective for teaching anatomy. More realistic models require access to more expensive 3D printing technologies and substantially longer design time, which would greatly increase the overall cost. Choosing an appropriate image acquisition modality is key. From a pedagogical viewpoint, 3DPAMs are effective tools for teaching anatomy, positively impacting the learning outcomes and satisfaction level. The pedagogical effectiveness of 3DPAMs seems to be best when they reproduce complex anatomical areas, and they are used by students early in their medical studies.

## Introduction

Practiced since Ancient Greece on animals, cadaver dissection is one of the main methods used to teach anatomy. Cadaveric dissection, carried out during hands-on training, supports the theoretical lessons given to medical students in universities and is currently considered the gold standard for learning anatomy [1–5]. However, there are many obstacles to using human cadaveric specimens, prompting a search for new pedagogical tools [6, 7]. Some of these new tools are extended reality, digital tools, and 3D printing. According to a recent literature review by Santos et al. [8] on the value of these new technologies for teaching anatomy, 3D printing appears to be one of the most relevant resources both in terms of its educational value to students and the feasibility of its implementation [4, 9, 10].

3D printing is not new. The first patents related to this technology date back to 1984: A Le Méhauté, O De Witte and JC André in France and 3 weeks later, C Hull in the USA. Since then, this technology has undergone continuous development, and its use has spread to numerous fields. For example, NASA printed the first object outside the planet Earth in 2014 [11]. The medical field has also appropriated this new tool, thus reinforcing the desire to develop personalized medicine [12].

Many authors have demonstrated the pedagogical benefits of using 3D-printed anatomical models (3DPAM) for medical education [10, 13–19]. When it comes to teaching human anatomy, non-pathological and anatomically normal models are required. Several reviews have studied pathological models or training models for a medical/surgical procedure [8, 20, 21]. With the intention of developing a hybrid teaching model for human anatomy that incorporates new tools such as 3D printing, we carried out a systematic review to describe and analyse how 3D-printed objects made for teaching of human anatomy are created and how students evaluate the pedagogical contribution of these 3D objects.

## Materials and methods

This systematic review of the literature was conducted in June 2022 without time limitation using the PRISMA (Preferred Reporting Items for Systematic Reviews and Meta-Analyses) guidelines [22].

### Eligibility criteria

Inclusion criteria were all research papers dealing with 3DPAM in anatomy teaching/learning. Literature reviews, letters, or articles studying pathological models, animal models, archaeological models, and medical/surgical training models were excluded. Only articles published in English were selected. Articles without available online abstracts were excluded. Articles dealing with several models – at least one of which was anatomically normal or had trivial pathology that did not alter the pedagogical value – were included.

### Search strategy

A literature search was performed in the electronic PubMed database (National Library of Medicine, NCBI) to identify relevant studies published up to June 2022. The following search terms were used: education, school, learning, teaching, learn, teach, educational, three-dimensional, 3D, 3-dimensional, printing, printed, print, anatomy, anatomical, anatomically, and anatomic. A single query was carried out: (((education[Title/Abstract] OR school[Title/Abstract] OR learning[Title/Abstract] OR teaching[Title/Abstract] OR learn[Title/Abstract] OR teach[Title/Abstract] OR educational[Title/Abstract]) AND (three dimensional[Title] OR 3D[Title] OR 3 dimensional[Title])) AND (printing[Title] OR printed[Title] OR print[Title])) AND (anatomy[Title/Abstract] OR anatomical[Title/Abstract] OR anatomically[Title/Abstract] OR anatomic[Title/Abstract]). Additional articles were identified through a manual search in the PubMed database and by looking through the references of other scientific articles. No date restriction was applied but the “human” filter was used.

### Study selection

All retrieved titles and abstracts were screened against the inclusion and exclusion criteria by two authors (EBR & AL), and any study that did not meet all the eligibility criteria were excluded. Full-text publications of the remaining studies were obtained and screened by three authors (EBR, EBE & AL). Any disagreement in the selection of articles was resolved, if necessary, by a fourth person (LT). Publications that met all the inclusion criteria were included in this review.

### Data extraction

Data extraction was performed independently by two authors (EBR & AL) and supervised by a third (LT).

The extracted data consisted of:

- study characteristics: publication date, country of authors, type of study
- model design data: anatomical region, specific anatomical part, initial model used for 3D printing, acquisition method, segmentation and modelling software, type of 3D printer, type and number of materials, printing scale, colours, cost of printing
- morphological evaluation of the model: model used for comparison, medical evaluation by an expert/teacher, number of raters, type of evaluation
- pedagogical performance of the 3D model: student knowledge assessment, assessment methods, number of students, number of comparison groups, randomization of students, type of education/student
- advantages and disadvantages.

All data were extracted in predefined forms.

## Results

### Study selection

**Fig. 1:**
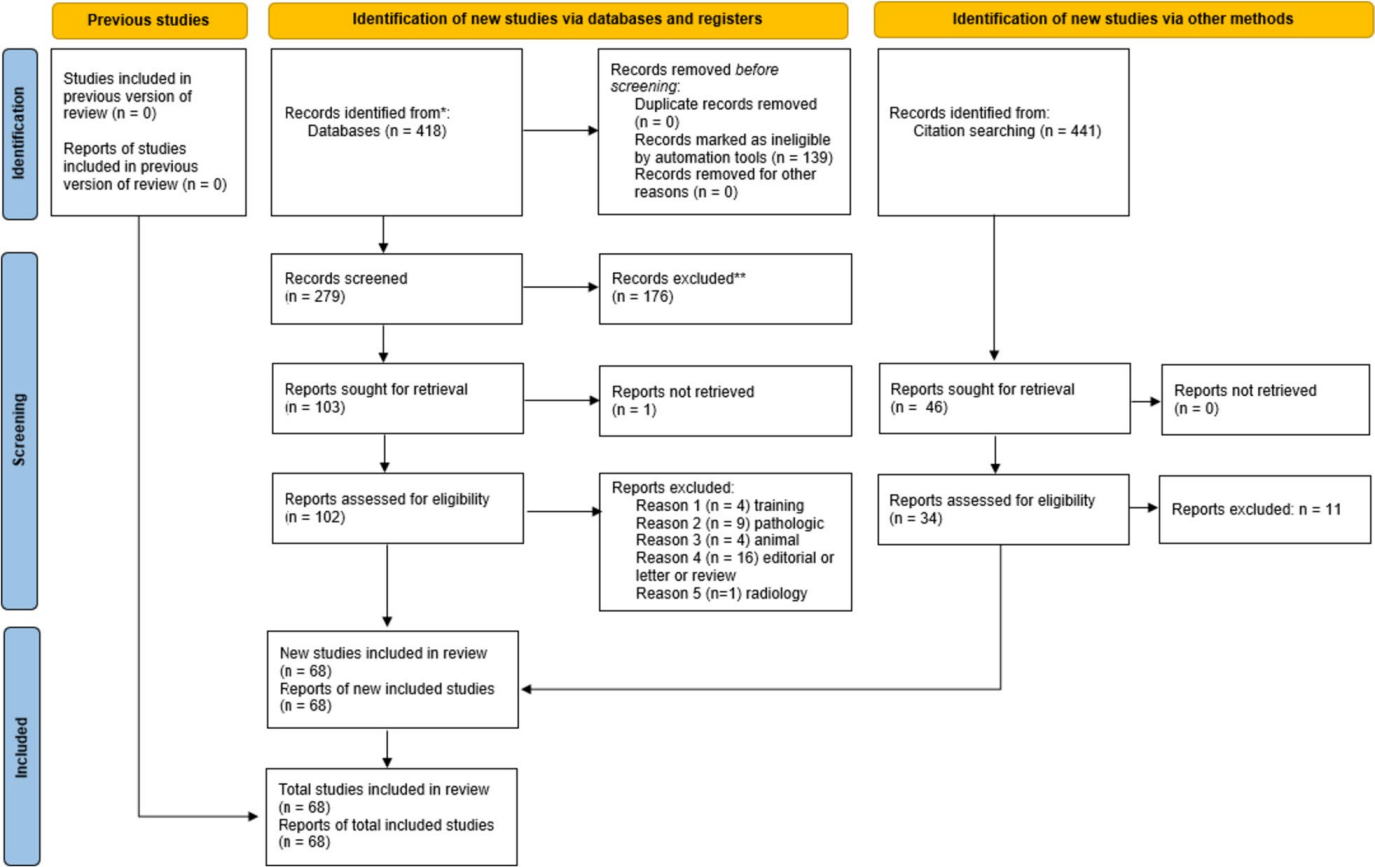
Flow diagram summarizing the identification, screening and inclusion of articles for this systematic review

Four hundred eighteen studies were identified in the MEDLINE database; 139 articles were excluded by the “human” filter. After the title and abstract were analysed, 103 studies were selected for reading of the full text. Thirty-four articles were excluded because they were either pathological models (9 articles), medical/surgical training models (4 articles), animal models (4 articles), 3D radiology models (1 article) or were not original scientific papers (16 articles). A total of 68 articles were included in this review. Figure 1 summarizes the selection process with a flowchart.

### Study characteristics

All studies were published between 2014 and 2022, with the average year of publication being 2019. Of the 68 articles included, 33 (49%) studies were descriptive and experimental, 17 (25%) were purely experimental and 18 (26%) were purely descriptive. Among the 50 (73%) experimental studies, 21 (31%) used randomization. Only 34 studies (50%) included a statistical analysis. Table 1 summarizes the characteristics of each study included.

**Table 1:** Summary of study characteristics

Author (Year)	Country	Type of study	Design of study	Description	Use of statistical tools
Ben Awadh et al. (2022) [23]	United Kingdom	Experimental	Randomized comparative controlled	3DPAM enhances novice learner interpretation of cross-sectional anatomy of the thorax	Y
Cercenelli et al. (2022)	Italy	Experimental and descriptive	Randomized	Educational tool evaluation combining 3DPAM and augmented reality	Y
Chandrasekaran et al. (2022) [24]	Singapore	Experimental	Randomized controlled cross-over	Validated instrument measuring students’ perceptions on plastinated and 3DPAM of the heart and the neck	Y
Hammerton et al. (2022) [25]	United Kingdom	Experimental		3DPAM acceptation for assessment by students and educators	N
Harmon et al. (2022) [26]	USA	Descriptive		3DPAM for health science students	N
Mogali et al. (2022) [27]	Singapore	Experimental	Randomized controlled cross-over	Effectiveness of 3DPAM compared to plastinated in learning cardiac and neck anatomy	Y
Saleh et al. (2022)	United Kingdom	Descriptive and experimental		Design of 3DPAM skull base, collaboration between clinicians and industry	N
Tan et al. (2022) [28]	China	Descriptive and experimental		Full color 3DPAM of the head and the upper limb	N
Bertolini et al. (2021) [29]	Italy	Descriptive and experimental		3DPAM of the heart	N
Krishnasamy et al. (2021) [30]	Malaysia	Descriptive and experimental		Heart 3DPAM rapid prototyping	N
Mahrous et al. (2021) [31]	USA	Descriptive and experimental		Comparison of instructional technologies: natural teeth, 3DPAM et augmented reality	Y
O’Brien et al. (2021) [32]	Canada	Experimental	Randomized controlled	Tracheo-bronchial 3DPAM to improve students understanding of segmentation anatomy	Y
Ruiz and Dhaher (2021) [33]	Italy and USA	Descriptive and experimental		Multi-color and multi-material 3DPAMs of knee joint	N
Smillie et al. (2021) [34]	United Kingdom	Descriptive and experimental		Producing 3DPAM of hepatobiliary system from CT imaging data	N
Vatankhah et al. (2021) [35]	Iran	Experimental	Randomized	3DPAM for teaching orbital anatomy	Y
Weatherall et al. (2021) [36]	Australia	Descriptive		3DPAM of pediatric airway models	N
Abdulcadir et al. (2020) [37]	Switzerland	Descriptive and experimental		3DPAM pelvic prototype to improve sexual anatomy and physiology	N
Chae et al. (2020) [38]	USA	Descriptive and experimental		Comparison between 3DPAM and 3D scanned temporal bone models	Y
Chedid et al. (2020) [39]	USA	Experimental	Randomized controlled cross-over	3DPAM of the liver helps learner identify hepatic subsegments	Y
Chen et al. (2020) [40]	China	Experimental	Randomized comparative	3DPAM improves residents’ understanding of gastrocolic trunk anatomy	Y
Damon et al. (2020) [41]	USA	Descriptive		Orientation planning of anatomical spine 3DPAM	N
Hojo et al. (2020) [42]	Japan	Descriptive and experimental		Utility of pelvic 3DPAM for lymph node dissection	Y
Javan et al. (2020) [43]	USA	Descriptive and experimental		3D visualization of pterygopalatine fossa using 3DPAM, serious game and virtual reality	N
Low et al. (2020) [44]	USA	Descriptive		Construction of frontal sinus 3DPAM	N
Radzi et al. (2020) [45]	Singapore	Descriptive and experimental		Heart 3DPAM for learning anatomy	Y
Tanner et al. (2020) [46]	USA	Descriptive and experimental	Randomized	Pterygopalatine 3DPAM enhances learning	Y
Tripodi et al. (2020) [47]	Australia	Descriptive		Impact of bones 3DPAM on first year students	Y
Williams et al. (2020) [48]	United Kingdom	Descriptive		High fidelity retroperitoneal 3DPAM	N
Backhouse al. (2019) [49]	Australia	Descriptive and experimental		3DPAM enables active and personalized learning	Y
Bartikian et al. (2019) [50]	Portugal	Descriptive and experimental		3DPAM of head bones	N
Cai et al. (2019) [51]	Singapore	Descriptive and experimental		Effects of knee joint 3DPAM in improving anatomical spatial knowledge	Y
Hojo et al. (2019) [52]	Japan	Experimental	Randomized controlled	Utility of pelvic 3DPAM for lateral pelvic lymph node dissection education	Y
Kanagasuntheram et al. (2019) [53]	Singapore	Descriptive		Composite midcarpal joint 3DPAM	N
Low et al. (2019)	USA	Descriptive and experimental	Randomized	Use of frontal sinus 3DPAM and 2D illustrations for resident education	Y
Shen et al. (2019) [54]	China	Descriptive		Process of skull 3DPAM for anatomy education	N
Skrzat et al. (2019) [55]	Poland	Descriptive and experimental		Temporal bone 3DPAM for teaching gross anatomy	N
Ugidos Lozano et al. (2019) [56]	Spain	Descriptive and experimental		Applicability of 3DPAM for students of health sciences	N
Yi et al. (2019) [57]	China	Experimental	Randomized controlled	Ventricular system 3DPAM in anatomy education	Y
Young et al. (2019)	Australia	Descriptive		3DPAM of archive human fetal material for teaching	N
Zhang et al. (2019) [58]	China	Descriptive and experimental		3DPAM for undergraduate medical students	Y
Bannon et al. (2018) [59]	Scotland	Descriptive		Pterygopalatine fossa negative 3DPAM	N
Casciato et al. (2018) [60]	USA	Descriptive		3DPAM to enhance cross sectional anatomy instruction	N
Garas et al. (2018) [61]	Australia	Experimental		3DPAM as a tool for anatomy education	Y
Mogali et al. (2018) [62]	Singapore	Descriptive and experimental		Evaluation by medical students of upper limb 3DPAM	Y
Smith C.F et al. (2018) [63]	United Kingdom	Experimental	Randomized controlled	3DPAM in undergraduate anatomy education	Y
Smith M.L et al. (2018) [64]	Ireland	Descriptive		3DPAM for anatomy education	Y
Suzuki et al. (2018) [65]	Japan	Descriptive		Transparent temporal bone and vestibulocochlear 3DPAM	N
Ugidos Lozano et al. (2018)	Spain	Descriptive		Different digitalization techniques for 3DPAM	N
Wu et al. (2018) [66]	China	Experimental	Randomized controlled	3DPAM enhance teaching and learning bone spatial anatomy	Y
Zhang et al. (2018) [67]	China	Descriptive and experimental	Randomized	Paranasal sinus 3DPAM	Y
Bücking et al. (2017) [68]	United Kingdom	Descriptive		From medical imaging to 3DPAM	N
Chen et al. (2017) [69]	China	Experimental	Randomized controlled	Role of skull 3DPAM in anatomy education	Y
Favier et al. (2017) [70]	France	Descriptive and experimental		Skull base 3DPAM for anatomical education and surgery simulation	N
Javan et al. (2017) [71]	USA	Descriptive		Cranial nerves 3DPAM	N
Kavanagh et al. (2017)	USA	Descriptive and experimental		Pediatric laryngeal simulator using 3DPAM	Y
Legocki et al. (2017) [72]	USA	Descriptive and experimental		Maxillofacial skeletal 3DPAM for entry-level	N
Lozano et al. (2017) [73]	Spain	Descriptive and experimental		Skull 3DPAM digitalization and prototyping	N
Fasel et al. (2016) [74]	Switzerland	Descriptive and experimental		Adapting anatomy teaching to surgical trends with classical dissection, 3DPAM and medical imaging	Y
Javan et al. (2016) [75]	USA	Descriptive		Understanding spatially complex anatomy with 3DPAM	N
Kong et al. (2016) [76]	China	Experimental	Randomized controlled comparative	3DPAM to improve teaching about hepatic segments to medical students	Y
Kong et al. (2016) [77]	China	Experimental	Randomized controlled	3DPAM to improve teaching about hepatic segments to medical students	Y
Lim et al. (2016) [16]	Australia	Experimental	Randomized controlled	Comparison between 3DPAM and cadaveric dissection for learning cardiac extern anatomy	Y
O’Reilly et al. (2016) [78]	Dublin	Descriptive and experimental	Randomized	Fabrication and assessment of lower limb et femoral vessel 3DPAM	Y
Shah et al. (2016) [79]	USA	Descriptive and experimental		Skull base 3DPAM to teach anatomy to neurosurgery residents	N
Adams et al. (2015) [80]	Australia	Descriptive and experimental		Orbital dissection 3DPAM reproduction for trainees in ophthalmology or optometry	N
Cohen et al. (2015) [81]	USA	Descriptive and experimental		Creation of temporal bone 3DPAM	N
Hochman et al. (2015) [82]	Canada	Descriptive and experimental	Randomized	Comparison between 3DPAM and virtual haptic temporal bone	Y
McMenamin al. (2014) [83]	Australia	Descriptive		Production of anatomical teaching resources using 3DPAM	N

*Abbreviations*: *3DPAM* 3D printed anatomical model, *N *no, *Y*  yes

### Model design data

Thirty-three articles (48%) studied the cephalic region, 19 (28%) the thoracic region, 17 (25%) the abdominopelvic region and 15 (22%) the limbs. Fifty-one articles (75%) mentioned 3D printing of bone as an anatomical model or within a multi-slice anatomical model.

Regarding the original model or file used for designing the 3DPAM, 23 articles (34%) mentioned the use of patient data, 20 articles (29%) the use of cadaver data, 17 articles (25%) the use of a database, and 7 studies (10%) did not disclose the origin of the file used.

In 47 studies (69%), the 3DPAMs were designed from CT scans, while 3 studies (4%) specified using micro-CT scans. In 7 articles (10%), the 3D objects were designed from optical scanners, in 4 articles (6%) from MRI and in 1 article (1%) from a camera and microscope. In 14 articles (21%), the origin of the source files for the design of the 3D model was not mentioned. The average spatial resolution was less than 0.5 mm for creating the 3D files. The best resolution was 30 µm [80] and the highest was 1.5 mm [32].

Sixty different software applications (segmentation, modelling, design, or printing) were used. Mimics (Materialise, Leuven, Belgium) was the most used (14 studies, 21%), followed by MeshMixer (Autodesk, San Rafael, CA) (13 studies, 19%), Geomagic (3D System, Morrisville, NC) (10 studies, 15%), 3D Slicer (Slicer Developer Orientation, Boston, MA) (9 studies, 13%), Blender (Blender Foundation, Amsterdam, The Netherlands) (8 studies, 12%) and CURA (Geldermalsen, The Netherlands) (7 studies, 10%).

Sixty-seven different printer models were mentioned with five printing processes. FDM (Fused Deposition Modelling) technology was used in 26 articles (38%), followed by material jetting in 13 articles (19%), then binder jetting (11 articles, 16%). Stereolithography (SLA) (5 articles, 7%) and selective laser sintering (SLS) (4 articles, 6%) were the least used technologies. The most used printer (7 articles, 10%) was the Connex 500 (Stratasys, Rehovot, Israel) [27, 30, 32, 36, 45, 62, 65].

When the material used to fabricate the 3DPAM was specified (51 articles, 75%), plastic and its derivatives were used in 48 (71%) studies. The main materials used were PLA (polylactic acid) (*n* = 20, 29%), resins (*n* = 9, 13%) and ABS (acrylonitrile butadiene styrene) (7 articles, 10%). Twenty-three articles (34%) studied 3DPAM made of several materials, 36 (53%) articles featured a 3DPAM made of only one material and 9 (13%) did not specify the material.

Twenty-nine articles (43%) mentioned the printing scale, which ranged from 0.25:1 to 2:1 and averaged 1:1. A 1:1 scale was used in 25 articles (37%). Twenty-eight 3DPAMs (41%) were composed of several colours and 9 (13%) were coloured after printing [43, 46, 49, 54, 58, 59, 65, 69, 75].

Thirty-four articles (50%) mentioned a cost. Nine articles (13%) mentioned the cost of the 3D printer and the raw materials. Printers ranged in price from 302 USD to 65,000 USD. The cost per model, when specified, ranged from 1.25 USD to 2800 USD; these extremes corresponded to a bone specimen [47] and a high-fidelity retroperitoneal model [48]. Table 2 summarizes the model design data for each included study.

**Table 2:** Summary of model design data

Author (Year)	Anatomical human region	Precise anatomical part	Initial model	Modality of acquisition	Type of software	Type of printer	Type of material (number)	Printing scale	Colors (number)	Cost of printing
Ben Awadh et al. (2022) [23]	Thorax	Heart	Patient and database	CT	3D Slicer, Blender, ideaMaker	FDM	NS	NS	N	NS
Cercenelli et al. (2022) [84]	Head	Dry skull, orbit	Cadaver	CT	D2P, Meshmixer	SLA	NS	NS	NS	NS
Chandrasekaran et al. (2022) [24]	Neck and thorax	Heart and neck	NS	NS		NS	NS	NS	N	NS
Hammerton et al. (2022) [25]	Thorax	Heart	Public database	NS	NS	NS	NS	NS	N	NS
Harmon et al. (2022) [26]	Upper and lower-limbs, pelvis, thorax	Bones	Public database	CT	In Vesalius, Meshmixer, Blender, Cura, 3D Slicer	FDM	NS	NS	NS	1.88 USD per model
Mogali et al. (2022) [27]	Thorax and neck	Full hearts, cross-section of the heart, coronary trees	Cadaver (plastinated human prosections)	CT	3D slicer, Materialise Magics	MJ	Photopolymers and translucent elastomers resins (Vero Yellow), Tango Plus and VeroMagenta	1 and 0.95	Y	6, 310, 319, 715 and 1,960 USD per model
Saleh et al. (2022)	Head	Temporal bone	Anonymized DICOM data	CT	Blender, MeshMixer, GrabCAD	MJ	Vero and Tango rigid and elastic polymers	1.4	Y	1300 GBP
Tan et al. (2022) [28]	Head, upper-limb	Skull, brain, face, hand muscles, blood vessels, nerves and deep structures	Database from frozen cadaver	optical	Maya	MJ	Hard and flexible	1	Y	NS
Bertolini et al. (2021) [29]	Thorax	Heart	Patient	CT	Mimics, 3-Matic (Materialise), Preform 3.3 (Formlabs), ScanStudio 2.0, CloudCompare (GPL)	SLA	Rigid and elastic resin	NS	N	NS
Krishnasamy et al. (2021) [30]	Thorax	Heart	Patient	CT	BioModroid	MJ and BJ	Rigid polymer, plastic and wax	1	N	NS
Mahrous et al. (2021) [31]	Head	Teeth	NS	NS		SLA	NS	1	N	NS
O’Brien et al. (2021) [32]	Thorax	Tracheobronchial tree	Patient	CT	3-Matic Medical	MJ	Vero clear	NS	N	NS
Ruiz and Dhaher (2021) [33]	Lower-limb	Knee joint	Previous study.stl file	NS	GrabCAD Print, Materialise 3-Matic (Materialise), SolidWorks, (Dassault), Rhinoceros 3D (Robert McNeel & Associates)	MJ	Agilus30, Tango and Digital ABS	NS	Y	NS
Smillie et al. (2021) [34]	Abdomen	Hepatobiliary system with stomach and duodenum	Database	Contrast enhanced CT	Simpleware ScanIP, Meshmixer, Blender, GrabCAD	MJ	VeroMagenta, VeroYellow, VeroCyan	NS	Y	1.343 GBP for raw plastic
Vatankhah et al. (2021) [35]	Head	Orbit	Patient	CT		NS	NS	NS	N	NS
Weatherall et al. (2021) [36]	Thorax	Airway with bone pieces	21-month-old patient and adult woman archived	CT	Avizo Lite, Geomagic studio 2014, Rhinoceros	BJ, MJ and SLA	White Version 3 resin, Flexible version 2 resin, VeroPure White, Tango Plus, Tango Black Polymers photopolymers, silicone elements	NS	Y	NS
Abdulcadir et al. (2020) [37]	Pelvis	Female pelvis with bone pieces	Patient	CT and MRI	Vitrea Vital Version 6.7.6 Canon, Kerne b40d, 3D Organ Segmentation (Vitrea), PrusaSlicer Version 2.0 (Slic3r), Blender	FDM	PLA	1 and 0.5	Y	NS
Chae et al. (2020) [38]	Head	Temporal bone	Cadaver	3D scan and micro-CT	Meshlab, iNtellect Cranial Navigation system, blender, 3D printer	SLA	resin	1	N	25 000 USD (optic scan), 200 USD (micro-CT), 3350 USD (SLA printer), 7 USD per model (resin)
Chedid et al. (2020) [39]	Abdomen	Liver segments	NS	CT		NS	NS	NS	Y	NS
Chen et al. (2020) [40]	Abdomen	Gastro-colic trunk	Patients	CT angiography	Advantage Workstation GE Medical System, Geomagic studio 2014 modelling software	NS	Thermoplastic urethane and resin	1	Y	NS
Damon et al. (2020) [41]	Vertebral column	Lumbosacral vertebrae	Database	NS	3D Slicer	FDM	ABS	NS	N	NS
Hojo et al. (2020) [42]	Pelvis	Pelvic lymph nodes with bone pieces	Patient	CT	Meshmixer version 3.5	FDM	PLA	NS	Y	15 USD
Javan et al. (2020) [43]	Head	Pterygopalatine fossa	Database	CT	Materialize InPrint, Autodesk 3D Studio Max 2018, Adobe Flash, iMaterialise.com	NS	PA and disk magnet	NS	AP	200 USD
Low et al. (2020) [44]	Head	Frontal sinus	Database	CT	Mimics, 3-matic (Materialise)	BJ	Gypsum powder	NS	Y	75.75 USD
Radzi et al. (2020) [45]	Thorax	Heart	Plastinated heart	CT	3D slicer, Materialise magics', Objet studio'	MJ	NS	1	Y	411 and 1639.7 Singapour dollar
Tanner et al. (2020) [46]	Head	Pterygopalatine fossa	Database	NS		FDM	PLA	NS	AP	NS
Tripodi et al. (2020) [47]	Upper-limb	Bones pieces	NS	NS	FlashPrint MeshMixer	FDM	PLA	0.25	N	635 USD (printer), 1.25 USD per model (filament)
Williams et al. (2020) [48]	Abdomen	Retroperitoneum with bone pieces	Patient	CT and MRI	Simpleware ScanIP, Meshmixer, Blender, GrabCAD	MJ	VeroMagenta, VeroYellow, VeroWhite	1	Y	2223.02£
Backhouse et al. (2019) [49]	Head	Orbit with bone pieces	Cadaver bone piece	3D scan	DAVID Laserscanner Pro Edition v4	NS	ABS	0.85	AP	2 USD per model, 5000 to 8000 USD (printer)
Bartikian et al. (2019) [50]	Head	Bone pieces	Cadaver bone piece	CT	3D Slicer, Craft-ware, Z-suite	FDM	PLA and ABS	NS	N	NS
Cai et al. (2019) [51]	Lower-limb	Knee joint	Patient	CT	InVesalius, Artec Studio, Rhino	SLS	PA EOS	1	N	90 USD per set
Hojo et al. (2019) [52]	Pelvis	Lateral pelvic lymph nodes with bone pieces	Patient	CT	Osirix MD Viewer, MeshMixer	FDM	NS	1	Y	NS
Kanagasuntheram et al. (2019) [53]	Upper-limb	Midcarpal joint	Patient	CT	Leios2 (Evatronix SA), Rhinoceros version 5	SLS	PA EOS and stainless steel with bronze	1	Y	150 USD per model (material)
Low et al. (2019)	Head	Frontal sinus	Patient	CT	Mimics (Materialise)	BJ	NS	NS	Y	NS
Shen et al. (2019) [54]	Head	Skull	Patient	CT	Mimics 17.0, Geomagic Studio (13.0), Autodesk Mudbox, MeshMixer	FDM	PLA	NS	AP	14 USD per model (material) and 500 USD (printer)
Skrzat et al. (2019) [55]	Head	Temporal bone	Cadaver bone piece	CT	In Vesalius, MeshLab	FDM	PLA and polyvinyl alcohol filament	1	N	NS
NSgidos Lozano et al (2019)	Head, thorax, small bones	Skull, small bones, vertebrae, thorax	NS	Laser scan	Geomagic	FDM	PLA	NS	N	NS
Yi et al. (2019) [57]	Head	Ventricular system with bone pieces	Patient	CT	PolyJet Studio 3DP	MJ	PLA and photocurable resin	NS	N	30 USD (material), “very expensive” printer
Young et al. (2019) [85]	Human gestational specimens	Week 4 to week 21	Fixed human embryonic and fetal specimens	CT	Mimics, version 17 (Materialise, Leuven, Belgium) 3D Coat, version 4.7.06 (Pilgway, Kiev, Ukraine), Stratasys 3D printing software PolyJet Studio,	BJ	gypsum-like plaster and binders, TangoPlus, VeroMagenta, yellow and cyan	1	Y	30 AUD USD (week 9 print) to 215 AUD USD (week 21 print)
Zhang et al. (2019) [58]	Head, neck, thorax, abdomen, pelvis, reproductive organ, lower-limb	Uterus, vagina, bladder, urethra, skin, cerebellum, brain stem, bone pieces, eyeball, optic nerve, liver, kidney, lung, penis	Database	Camera and microscope	Amira	FDM and SLA	PLA and photosensitive resin	NS	AP	NS
Bannon et al. (2018) [59]	Head	Pterygopalatine fossa	Database	NS	3D slicer, Meshmixer, Blender (open source), Slic3r Prusa Edition (G-code conversion)	FDM	PLA	1 and 2	AP	0.13£ (Scotland)
Casciato et al. (2018) [60]	Lower-limb	Right leg	Cadaver	NS	Microsoft PowerPoint, Selva3D, CURA	FDM	PLA	NS	N	304 USD (full cost) including 2.13 USD (filament) and printer
Garas et al. (2018) [61]	Trunk	Heart, shoulder, tight	NS	NS	NS	NS	NS	NS	Y	NS
Mogali et al. (2018) [62]	Upper-limb	Left upper-limb	Cadaver (plastinated)	CT	3D Doctor (Able Software)	MJ	soft elastomers and rigid plastic	NS	Y	390 USD
Smith et al. (2018) [64]	Thorax, musculo-skeletal	Respiratory system, musculo-skeletal system	Cadaver	CT	Materialize Mimics v15.0, MakerWare	FDM	ABS	From 0.4 to 1	N	NS
Smith et al. (2018) [63]	Head and neck	Posterior triangle of the neck, laryngeal model, intrinsic muscles of the larynx and vocal folds, bone pieces	Database	NS	MeshMixer, CURA LulzBot	FDM	PLA and FilaFlex	NS	Y	4077 USD (printer); 26 USD (FilaFlex), 40 USD (PLA). 144 to 387 USD per model
Suzuki et al. (2018) [65]	Head	Temporal bone, vestibulocochlear organ	Cadaver	CT	Synapse Vincent (Fujifilm)	MJ	Ultraviolet transparent curing resin	0.66	AP	NS
Ugidos Lozano et al. (2018)	Head and arm	Bone pieces	Cadaveric bone	CT and laser scan	3D slicer, Artec studio 3D, Geomagic Design X 3D, CURA	FDM	PLA	1	N	NS
Wu et al. (2018) [66]	Spine, pelvis, upper and lower-limbs	Bone pieces	Patient	CT	Mimics 16.0	NS	NS	NS	NS	6 to 10.50 USD (spine and limbs) to 90 USD (pelvis)
Zhang et al. (2018) [67]	Head	Sinus-skull base	Patient	CT	Mimics, Cura version 15.02	FDM	PLA	1	N	3 USD
Bücking et al. (2017) [68]	Thorax and abdomen	Ribs, lung, liver	Database	NS	Mimics, Simpleware, Freesurfer, Seg3D, 3D Slicer, MeshMixer, Cura, InVesalius, ITK Snap, Osirix Lite, Horos, ImageJ	FDM	PLA	NS	N	16£ (lung), 25£ (ribs), 10£ (liver)
Chen S. et al. (2017) [69]	Head	Skull	Cadaveric bone	CT	Mimics, Geomagic, 3ds Max, MeshMixer	FDM	PLA	NS	AP	500 USD (printer) and 14 USD (material)
Favier et al. (2017) [70]	Head	Bone pieces	Cadavers	CT and micro-CT	Medical Image Segmentation Tool, Meshlab	MJ and BJ and SLS and FDM	Multicolor plaster, resin, PA, polycarbonate, composite powder	1	N	155 USD (computer), 275 USD (Multicolor), 880 USD (resin), 298 USD (PA)
Javan et al. (2017) [71]	Head	Cranial nerve, brainstem, skull	Patient	NS	Materialise Mimics, Adobe Photoshop, Osirix Lite, Autodesk 3D Studio Max	NS	PA	1.2	N	120 USD
Kavanagh et al. (2017)	Neck and thorax	Larynx and trachea	NS	CT	SolidWorks (Dassault Systèmes)	FDM	PLA, ABS, HIPS	NS	N	1.75 USD to 4.66 USD per model
Legocki et al. (2017) [72]	Head	Skull, mandible and maxilla	Patient	CT	Osirix, MeshLab	FDM	PLA and thermoplastic	1	N	1000 USD including 90.85 to 91.65 USD per model and 2 × 22.5 USD per hour and excluding 3598 USD for printer and software
Lozano et al. (2017) [73]	Head	Skull	Cadaver	3D scan	Geomagic design 3D, Repetier-Host, CURA (Ultimaker trading house)	FDM	PLA	1	N	“Low cost”
Fasel et al. (2016) [74]	Head, neck, thorax, abdomen and pelvis	Isthmus of the thyroid, diaphragm and aorta	Cadaver	CT	Osirix, Mimics 14.12	BJ	NS	1	N	NS
Javan et al. (2016) [75]	Head, thorax, abdomen and pelvis	Liver, lung, prostate, coronary arteries, circle of Willis	Database	MRI	Autodesk 3D Studio Max, Osirix lite, Slicer	NS	PA	NS	AP	40 to 100 USD per model
Kong et al. (2016) [76]	Abdomen	Liver	Cadaver	CT	Mimics, Geomagic, Zedit 3.21 (3D system)	Ink-jet 3DP	composite powder, curing agent, transparent jelly wax	NS	Y	600 USD per model
Kong et al. (2016) [77]	Abdomen	Liver	Patient	CT	3DV system, Zedit 3.21	Ink-jet 3DP	composite powder, curing agent	NS	Y	NS
Lim et al. (2016) [16]	Thorax	Heart	Cadaver (prosection)	CT	Avizo Lite, 3D coat version	BJ	NS	1	Y	NS
O’Reilly et al. (2016) [78]	Lower-limb	Bone, tendon, muscles	Database	NS	MeshLab, version 1.3.3, Tinkercad (Autodesk Inc., San Francisco, CA), Z EditTM Pro, (3D Systems Corp., Rock Hill, SC)	BJ	Composite powder and silicone	0.5 and 0.33	Y	NS
Shah et al. (2016) [79]	Head	Sphenoid sinus	Patient	MRI and CT	NC (3-D printing system)	NS	Thermoplastic	NS	N	NS
Adams et al. (2015)	Head	Orbit with bone pieces	Cadaver	Laser scan	Artec studio Version9.0, 3D Coat (PILGWAY, Ukraine), Geomagic (3D systems, USA)	BJ	Composite powder and powdered plastic	1	Y	NS
Cohen et al. (2015) [81]	Head	Temporal bone	Patient	CT	ITK-SNAP	FDM	ABS	NS	Y	27.61 to 42.02 USD
Hochman et al. (2015) [82]	Head	Temporal bone	Cadaver	Micro-CT	Mimics 14.0, Geomagic	NS	remnant material, binding agent	NS	Y	NS
McMenamin et al. (2014) [83]	Upper-limb	Wrist and hand	Cadaver (prosection)	CT	Avizo Lite, 3D coat version	BJ	NS	1	Y	65 000 USD (printer), 8000 USD (software), 400 USD per hour (CT), 5000 USD (computer), 0.55 USD per cc (consumables) and 40,000 USD (technical staff)

*Abbreviations*: *AP* A posteriori, *BJ*  Binder jetting, *ABS* Acrylonitrile butadiene styrene, *CT* Computed tomography, *FDM* Fused deposition modelling, *GBP* Great Britain Pound, *HIPS* High impact polystyrene, *micro-CT* Micro-computed tomography, *MJ* Material jetting, *MRI* Magnetic Resonance Imaging, *N* No, *NS* Not specified, *PA* Polyamide, *PLA* Polylactic acid, *SLA* Stereolithography, *USD* United States dollars, *Y* Yes

### Morphological evaluation of 3D models

Thirty-seven studies (54%) compared the 3DAPM to a reference model. Among these studies, the most common comparator was a reference anatomical model, which was used in 14 articles (38%), a plastinated specimen in 6 articles (16%), virtual reality in 6 articles (16%), CT-scan imaging in 5 articles (14%), another 3DPAM in 3 articles (8%), a serious game in 1 article (3%), radiographs in 1 article (3%), a business model in 1 article (3%), and augmented reality in 1 article (3%). Thirty-four (50%) studies rated the 3DPAM. Fifteen (48%) studies specified the raters’ experience (Table 3). The 3DPAM was evaluated by surgeons or attending physicians in 7 studies (47%), anatomy experts in 6 studies (40%), students in 3 studies (20%), teachers (without specifying the discipline) in 3 studies (20%) and another rater in 1 article (7%). The average number of raters was 14 (minimum 2, maximum 30). The morphology of the 3DPAM was evaluated qualitatively in 33 studies (49%) and quantitatively in 10 studies (15%). Among the 33 studies using a qualitative assessment, 16 studies used a purely descriptive assessment (48%), 9 studies used tests/scores/surveys (27%) and 8 studies used a Likert scale (24%). Table 3 summarizes the morphological evaluation of the models in each included study.

**Table 3:** Summary of how the morphology of the 3D models was evaluated

Author (Year)	Model used for comparison	Qualification of evaluator (number)	Type of evaluation
Ben Awadh et al. (2022) [23]	2D images	NS	NS
Cercenelli et al. (2022) [84]	3DPAM and VR versus 2D images	NS	NS
Chandrasekaran et al. (2022) [24]	Plastinated	NS	NS
Hammerton et al. (2022) [25]	NS	Anatomy senior (2) and educators (11)	Qualitative
Mogali et al. (2022) [27]	Plastinated	NS	NS
Saleh et al. (2022)	NS	Authors (5)	Qualitative
Tan et al. (2022) [28]	Cadaver and digital	Anatomist (5) and surgeons (3)	Qualitative and quantitative
Bertolini et al. (2021) [29]	CT images	Authors (3)	Qualitative and quantitative
Krishnasamy et al. (2021) [30]	NS	Surgeons, cardiologists, radiologists, surgical registrars (30)	Survey
Mahrous et al. (2021) [31]	Natural tooth, 3D AR and VR	NS	NS
O’Brien et al. (2021) [32]	2D images	NS	NS
Ruiz et al. (2021) [33]	NS	Authors (2)	Quantitative
Weatherall et al. (2021) [36]	NS	Authors (6)	Qualitative
Abducaldir et al. (2020)	2D diagrams	Expert researchers (30)	Semi-structure interview
Chae et al. (2020) [38]	Cadaver (temporal bone), optic scanner and micro-CT images	Authors (8)	Quantitative
Chedid et al. (2020) [39]	2D images	NS	NS
Chen et al. (2020) [40]	2D images	NS	NS
Damon et al. (2020) [41]	Same model with and without initial rotation	NS	NS
Hojo et al. (2020) [6]	3D VR and CT images	Surgeons (30)	Likert and Adachi classification
Javan et al. (2020) [43]	Serious gaming and VR	NS	NS
Low et al. (2020) [44]	NS	Authors (4)	Qualitative
Radzi et al. (2020) [45]	Plastinated	NS	NS
Tanner et al. (2020) [46]	Cadaver (half-skull)	NS	NS
Bartikian et al. (2019) [50]	Same model with different printer	Authors (4)	Qualitative
Cai et al. (2019) [51]	Cadaver (knee skeleton)	Experts in human anatomy (2)	Qualitative
Low et al. (2019)	2D images	NS	NS
Shen et al. (2019) [54]	Cadaver (skull) (other study)	NS	NS
Skrzat et al. (2019) [55]	Cadaver	Authors (4)	Qualitative
Ugidos Lozano et al. (2019) [56]	2D images and cadaver (bones)	NS	NS
Yi et al. (2019) [57]	NS	Professor of anatomy (2) and professor of surgery (2)	Likert
Zhang et al. (2019) [58]	NS	Experienced teachers (5)	Scores
Casciato et al. (2018) [60]	NS	Authors (3)	Quantitative
Garas et al. (2018) [61]	Cadaver and plastinated	NS	NS
Mogali et al. (2018) [62]	Plastinated	Students (15)	Qualitative
Smith et al. (2018) [64]	Cadaver (teacher) and 2D images (students)	Teachers (6)	Survey
Smith et al. (2018) [64]	NS	Authors (2)	Qualitative
Suzuki et al. (2018) [65]	NS	Authors (9)	Qualitative
Ugidos Lozano et al. (2018)	NS	Authors (6)	Qualitative
Wu et al. (2018) [66]	Radiographics	NS	NS
Zhang et al. (2018) [67]	CT images	Senior doctors (9)	Survey and Likert
Chen et al. (2017) [69]	2D images and cadaver	Students (26)	Likert
Favier et al. (2017) [70]	Cadaver	Authors (9)	Quantitative
Javan et al. (2017) [71]	NS	Authors (3)	Qualitative and descriptive
Kavanagh et al. (2017)	NS	Authors (6)	Quantitative and Likert
Legocki et al. (2017) [72]	Commercial model	Authors (3)	Quantitative and qualitative
Fasel et al. (2016) [74]	CT images and cadaver (dissection)	Students (12)	Quantitative
Khong et al. (2016)	3 different 3DPAM and 2D images	Anatomy teachers (4) and consultants of surgery (2)	Likert
Khong et al. (2016)	3D VR and 2D images	Anatomy teachers (4) and consultants of surgery (2)	Likert
O’Reilly et al. (2016) [78]	Cadaver	NS	NS
Shah et al. (2016) [79]	2D images	NS	NS
Adams et al. (2015) [80]	NS	Authors (6)	Satisfaction
Cohen et al. (2015) [81]	NS	Authors (2)	Qualitative
Hochman et al. (2015) [82]	3D VR	NS	NS
McMenamin et al. (2014) [83]	Plastinated	Authors (4)	Descriptive

*Abbreviations*: *3DPAM* 3D printed anatomical model, *AR *Augmented reality, *CT *Computed tomography, *NS* Not specified, *VR* Virtual reality

### Pedagogical performance of 3D models

Thirty-three (48%) articles investigated and compared the pedagogical performance of 3DPAMs in students. Among these studies, 23 (70%) articles evaluated student satisfaction, 17 (51%) used a Likert scale and 6 (18%) used other methods. Twenty-two articles (67%) evaluated student learning through a knowledge check, 10 (30%) of which administered pre- and/or post-tests. Eleven studies (33%) used multiple-choice questions and quizzes to assess students' knowledge and 5 (15%) used image labelling/anatomical identification. An average of 76 students participated per study (minimum 8, maximum 319). Twenty-four studies (72%) had comparison groups, 20 (60%) of which applied randomization. Conversely, 1 study (3%) randomized the anatomical models to assign them to 10 different students. On average, 2.6 groups were compared (minimum 2, maximum 10). Twenty-three studies (70%) involved medical students, of which 14 (42%) included first-year students. Six (18%) studies involved residents, 4 (12%) dental students, and 3 (9%) science students. Six studies (18%) implemented and evaluated self-directing learning with the 3DPAM. Table 4 summarizes how the pedagogical performance of 3DPAMs was evaluated in each included study.

**Table 4:** Summary of how the pedagogical performance of 3D models was evaluated

Author (Year)	Knowledge assessment	Knowledge assessment method	Number of student / Number of comparison groups	Randomization	Type of education/student	Satisfaction evaluation	Satisfaction assessment method
Ben Awadh et al. (2022) [23]	Y	Pre and posttest (cross-section images labelling questionnaire and mental rotation test)	319 / 2	N	1st year medical students	Y	Likert
Cercenelli et al. (2022) [84]	Y	MCQ and practical task	62/2	Y	second-year medical students	Y	Likert
Chandrasekaran et al. (2022) [24]	N	NS	96/2	Y	1st year medical students	Y	Likert
Hammerton et al. (2022) [25]	N	NS	84 / x	N	1st year medical students	Y	Likert (semi-structured interview)
Harmon D.J. et al. (2022) [26]	N	NS	80 / x	N	2^nd^ year doctors, 1st year medical and dental students	Y	Qualitative
Mogali et al. (2022) [27]	Y	Qualtrics software pre-test (MCQ)	63 / 2	Y	1st year medical students	N	
Mahrous et al. (2021) [31]	N	NS	70 / x	N	Dental students	Y	Survey
O’Brien et al. (2021) [32]	Y	MCQ (structure identification on cross-sectional images) immediate and delayed test	31 / 2	Y	1st year medical students	N	
Vatankhah et al. (2021) [35]	Y	Pre and post-test MCQ	13 / 2	Y	1^st^ and 2^nd^ year residents	N	
Chedid et al. (2020) [39]	Y	Test questions	116 / 2	Y	Gastro-enterology, radiology and general surgery departments	N	
Chen et al. (2020) [40]	Y	Pre and post-test	47 / 2	Y	Residents	Y	Questionnaire
Radzi et al. (2020) [45]	N	NS	58 / x	N	1st year medical students	Y	Likert
Tanner et al. (2020) [46]	Y	Quiz with pre and post test	123 / 2	Y	Junior and sophomores in medical education, premedecine undergraduate, graduate students of Master of Biomedical Science and 1st year medical students, dental and physicians’ students	Y	NS
Tripodi et al. (2020) [47]	N	NS	111 / x	N	1st year osteopathy students	Y	Likert and long answer survey
Backhouse et al. (2019) [49]	N	NS	81 / x	N	1st year students in ocular anatomy unit bachelor or vision sciences and master of optometry	Y	Likert
Cai et al. (2019) [51]	Y	MCQ	35 / 2	Y	1st year medical students	N	
Hojo et al. (2019) [52]	Y	Short and long tests	102 / 2	Y	Medical students, residents and one colorectal surgeon	Y	Likert
Low et al. (2019)	Y	Pre and post-tests	41 / 2	Y	Residents (radiology and ENT)	N	
Ugidos Lozano et al. (2019) [56]	N	NS	280 / x	N	Physiotherapy, medicine, nursing, occupational therapy and dentistry students of Health Sciences	Y	Likert
Yi et al. (2019) [57]	Y	Pre and post-tests (theorical and practical questions)	60 / 3	Y	2nd year medical students	Y	Likert
Zhang et al. (2019) [58]	Y	Reports	30 / 10	Y	5 year medical students	Y	Likert
Garas et al. (2018) [61]	Y	9 questions test (identify pinned structures)	23 / 2	N	1st year health sciences and 3rd year Human Biology Preclinical students	Y	Likert
Mogali Et al. (2018)	N	NS	15 / x	N	2nd year medical students	Y	Likert
Smith et al. (2018) [63]	Y	Test questions	127 / 2	Y	1st year medical students	Y	Key themes from focus group
Wu et al. (2018) [66]	Y	Questions	90 / 2	Y	Medical students completed anatomy courses	Y	Likert
Chen et al. (2017) [69]	Y	MCQ and labelled structures to be recognized	79 / 3	Y	3^rd^ year medical students	Y	Likert
Fasel et al. (2016) [74]	N	NS	12 / x	N	Undergraduate medical students	Y	Scale 1 to 6
Kong et al. (2016) [77]	Y	Quiz	92 / 4	Y	1st year medical students	N	
Kong et al. (2016) [77]	Y	Quiz	61 / 3	Y	1st year medical students	N	
Lim et al. (2016) [16]	Y	MCQ and labelled structures to be recognized pre and post-tests	53 / 3	Y	1st year medical students	N	
O’Reilly et al. (2016) [78]	Y	Quizdom System pre and post-test	22 / 2	Y	Graduate entry medicine year 1 class	Y	Likert
Shah et al. (2016) [79]	Y	Labelled structures to be recognized	8 / 2	N	Neurosurgery residents (junior and senior)	N	
Hochman et al. (2015) [82]	N	NS	10 / 10	Y	Residents	Y	Likert

*Abbreviations*: *ENT* Ear nose throat, *MCQ* Multiple choice question, *N* No, *NS* Not specified, *Y* Yes

### Advantages and disadvantages

The main advantages reported by the authors using 3DPAM as a pedagogical tool for teaching normal human anatomy were the visual and haptic characteristics, including authenticity [55, 67], precision [44, 50, 72, 85], variability of consistencies [34, 45, 48, 64], colours and transparency [28, 45], solidness [24, 56, 73], effectiveness for education [16, 32, 35, 39, 52, 57, 63, 69, 79], cost [27, 41, 44, 45, 48, 51, 60, 64, 80, 81, 83], reproducibility [80], possibility of improvement or personalization [28, 30, 36, 45, 48, 51, 53, 59, 61, 67, 80], possibility of manipulation by the students [30, 49], time savings for teaching [61, 80], ease of storage [61], possibility of integrating functional anatomy or creating a specific design [51, 53, 67], rapid design for bone models [81], possibility of co-creation and taking the model home [49, 60, 71], improvement in mental rotation ability [23] and knowledge retention [32], and positive effect on educators [25, 63] as well as student satisfaction [25, 45, 46, 52, 52, 57, 63, 66, 69, 84].

The main drawbacks were related to design: stiffness [80], consistency [28, 62], lack of detail or transparency [28, 30, 34, 45, 48, 62, 64, 81], overly bright colours [45], and fragility [71]. Other drawbacks were the loss of information [30, 76], long time needed for image segmentation [36, 52, 57, 58, 74], printing time [57, 63, 66, 67], lack of anatomical variability [25] and the high cost [48].

## Discussion

This systematic review summarizes 68 articles published over 9 years, highlighting the scientific community’s interest in 3DPAM as a pedagogical tool for teaching normal human anatomy. Every anatomical region has been studied and printed in 3D. Among these articles, 37 compared the 3DPAM to another model and 33 evaluated the pedagogical relevance of the 3DPAM for students.

Given the differences in the design of studies on 3D printing in anatomy, we did not feel it was appropriate to carry out a meta-analysis. A meta-analysis published in 2020 focused mainly on post-training tests of anatomical knowledge, without analysing the technical and technological aspects of the design and manufacture of 3DPAMs [10].

### Model design data

The cephalic region was the most studied, probably because its anatomical complexity makes it difficult for students to picture this anatomical region in 3D space, compared to the limbs or trunk. CT scan was by far the most used image acquisition modality. This modality is widely available, especially in healthcare facilities, but its spatial resolution is limited, and its soft-tissue contrast is low. These limitations make CT scan unsuitable for segmentation and modelling of the nervous system for example. On the other hand, CT scan was preferred for the segmentation/modelling of bone tissue; the bone/soft tissue contrast facilitates these steps before 3D printing of an anatomical model. Micro-CT, on the other hand, was cited as the reference technology in terms of spatial resolution for the acquisition of bone tissue images [70]. An optical scanner or MRI can also be used for image acquisition. Higher resolution prevents the smoothing of bone surfaces and preserves the subtleties of the anatomy [59]. The choice of models also influences the spatial resolution; for example, plastinated models have lower resolution [45]. A graphic designer was needed when creating highly customized 3D models, which increases the cost (25 to 150 USD per hour of work) [43]. Obtaining a good quality.STL file was not sufficient to produce a good quality anatomical model. The printing parameters such as the orientation of the anatomical model on the printing plate must be defined [29]. Some authors suggested that advanced printing technologies such as SLS should be used whenever possible to improve the 3DPAM’s accuracy [38]. The help of a professional was required to make the 3DPAM; the most requested professionals were an engineer [72], radiologist, [75] graphic designer, [43] and anatomist [25, 28, 51, 57, 76, 77].

Segmentation and modelling software are important factors for obtaining an accurate anatomical model, but the price of these software packages and their complexity hinder their use. Some studies compared the use of different software packages and printing technologies, highlighting the advantages and disadvantages of each [68]. In addition to modelling software, printing software is required that is compatible with the chosen printer; some authors preferred to use online 3D printing [75]. If enough 3D objects will be printed, the investment may be financially profitable [72].

Plastic was by far the most used material. It is the material of choice for 3DPAM due to its large range of textures and colours. Several authors praised its high strength compared to traditional cadaveric or plastinated models [24, 56, 73]. Some plastics even have flexural or tensile properties. For example, the Filaflex used with FDM technology can stretch up to 700%. For some authors, it is the material of choice for reproducing muscles, tendons and ligaments [63]. On the other hand, two studies raised questions about the direction of the fibres as printed. Indeed, the direction of the muscle fibres is critical when modelling a muscle, along with its insertions, innervation and function [33].

Surprisingly, few studies mentioned the printing scale. Since many consider a 1:1 scale as standard, the authors may have decided not to mention it. The possibility of enlargement has not been explored much despite its benefit for directed teaching in large groups, especially given the increasing number of students per class where the actual size of the model is an important element. Of course, a full-size scale makes it easier to locate the various anatomical elements and to transpose it to patients, which probably explains why this scale is often used.

Among the multiple printers available on the market, those that provide high-definition printing in colour and in several materials – thus several textures – using PolyJet technology (material jetting or binder jetting) cost between 20,000 and 250,000 + dollars (https://www.aniwaa.com/). This high cost likely restricts the diffusion of 3DPAMs in medical schools. In addition to the price of buying a printer, the materials needed for material jetting cost more than those used for SLA or FDM printers [68]. The price of SLA or FDM printers is also more manageable, ranging from 576 to 4999 € in the articles listed in this review. According to Tripodi and colleagues, bone parts could be printed for 1.25 USD each [47]. Eleven studies concluded that 3D printing costs less than plastinated or commercial models [24, 27, 41, 44, 45, 48, 51, 60, 63, 80, 81, 83]. Furthermore, these commercial models are intended for patient information and do not have sufficient detail to be used for teaching anatomy [80]. These commercial models were considered inferior to 3DPAMs [44]. It is important to note that – in addition to the printing technology used – the final cost is also proportional to the scale and thus the final size of the 3DPAM [48]. For these reasons, the preferred scale was full size [37].

### Morphological evaluation of 3D models

Only one study compared a 3DPAM to a commercially available anatomical model [72]. Cadaveric specimens were the most used comparator for 3DPAM. Despite its drawbacks, the cadaveric model remains a valuable tool for teaching anatomy. A distinction needs to be made between cadaveric dissection, prosections and dry bones. Two studies found that 3DPAMs were significantly more effective than plastinated prosections based on learning tests [16, 27]. A single study compared one hour of learning using a 3DPAM (lower limb) with one hour of dissection on the same anatomical area [78]. There was no significant difference between the two teaching methods. It is likely that few studies have been done on this topic because this comparison is difficult to set up. Dissection by students is a time-consuming task to prepare for. Several dozens of hours of dissection are sometimes necessary, depending on the dissection subjects. A third comparison can be made with dry bones. The studies by Cai and Smith found significantly better test results for the groups who used 3DPAM [51, 63]. Chen and colleagues specified that students who used the 3D model were better at recognizing structures (skull) but that there was no difference in MCQ results [69]. Finally, Tanner and colleagues demonstrated better post-test results for the group using a 3DPAM of the pterygopalatine fossa [46]. This literature review identified other new teaching tools. Among the most common were augmented reality, virtual reality, and serious gaming [43]. According to Mahrous and colleagues, the anatomical model preference depends on the number of video game hours played by the student [31]. On the other hand, the main pitfall of new tools in anatomy education is haptic feedback, especially for virtual-only tools [48].

### Pedagogical performance of 3D models

A knowledge pre-test was used in most studies evaluating new 3DPAMs. These pre-tests help to avoid assessment bias. Some authors excluded all students who scored above average on the pre-test before conducting their experimental study [40]. Among the assessment biases, Garas and colleagues cited the colouring of the models but also the choice of volunteers among the student classes [61]. Staining makes anatomical structures easier to identify. Chen and colleagues imposed strict experimental conditions, with no initial intergroup differences and as much blinding as possible [69]. Lim and colleagues suggest avoiding assessment bias by having the post-test assessment prepared by a third person [16]. Some of the studies used Likert scales to assess the 3DPAM’s appropriateness. This tool is suitable for evaluating satisfaction but nevertheless has important biases that one must be aware of [86].

The educational relevance of 3DPAMs was evaluated mostly in medical students, including first-year students in 14 of the 33 studies identified. In their pilot study, Wilk and colleagues reported that medical students felt 3D printing should be incorporated into their learning of anatomy [87]. Eighty-seven percent of students surveyed in the Cercenelli study felt that their second year was the best time to use 3DPAMs [84]. Results from Tanner and colleagues also showed that students were better if they had never studied the area [46]. These data suggest that the first years of medical school are the best time to incorporate 3DPAMs into the teaching of anatomy. Ye's meta-analysis corroborates this idea [18]. Of the 27 articles included in their study, there was a significant difference in test results in favour of 3DPAMs versus conventional models for medical students but not for residents.

3DPAMs were effective as pedagogical tools in terms of achievement, [16, 35, 39, 52, 57, 63, 69, 79] long-term knowledge retention [32] and student satisfaction [25, 45, 46, 52, 57, 63, 66, 69, 84]. Expert panels have also been found these models useful [37, 42, 49, 81, 82] and two studies highlighted teacher satisfaction with 3DPAMs [25, 63]. Among all resources, Backhouse and colleagues judged 3D printing to be the best alternative to conventional anatomical models [49]. In their first meta-analysis, Ye and colleagues affirm that the post-test results of students who received instruction incorporating 3DPAMs were better than those who received instruction in 2D or on a cadaver [10]. However, they did not differentiate the 3DPAMs by their complexity but simply as heart, nervous system and abdomen. In seven studies, 3DPAMs were not superior to other models based on the knowledge tests given to students [32, 66, 69, 77, 78, 84]. In their meta-analysis, Salazar and colleagues conclude that the use of 3DPAMs specifically improves the understanding of complex anatomical structures [17]. This concept is consistent with a letter to the editor by Chytas [88]. Certain anatomical areas that are considered less complex would not require the use of 3DPAMs, while more complex anatomical areas such as the neck or nervous system would be a reasonable choices for 3DPAMs. This notion probably explains why some 3DPAMs have not been judged superior to conventional models, especially since the model's effectiveness seems to be better when the student has no knowledge in the field. Consequently, a simple model, presented to students who already have some knowledge of the subject (advanced medical students or residents), would be useless for improving student results.

### Advantages and disadvantages

Of all the educational benefits listed, 11 studies highlighted the visual or tactile qualities of their models, [27, 34, 44, 45, 48, 50, 55, 63, 67, 72, 85] while 3 studies emphasized the strength and durability (33, 50–52, 63,79,85,86). Other advantages were that the students could manipulate the structures, the teacher could save time, they were easier to preserve than a cadaver, the design could be completed in less than 24 h, it could be used as a home study tool and it could be used to teach large groups [30, 49, 60, 61, 80, 81]. The 3D printing of multiple copies for teaching anatomy in large groups, make the 3D printing of models more cost-effective [26]. Using 3DPAMs increased mental rotation ability [23], and improved interpretation of cross-sectional imaging [23, 32]. Two studies found that students exposed to 3DPAMs were more attracted to surgery [40, 74]. Metal connectors can be incorporated to produce the motion needed to study functional anatomy [51, 53] or to print the model with a page-turning design [67].

3D printing made it possible to create adjustable anatomical models by improving certain aspects during the modelling stage, [48, 80] creating a suitable base, [59] merging multiple models, [36] using transparency, (49) colour, [45] or making certain internal structures visible [30]. Tripodi and colleagues used modelling clay to supplement their 3D printed bone models, highlighting the value of co-creating the model as a teaching tool [47]. In 9 studies, colour was applied after printing, [43, 46, 49, 54, 58, 59, 65, 69, 75] but only once by the students [49]. Unfortunately, that study did not assess the pedagogical quality of the model or the teaching sequence. This is something to take into consideration in the context of anatomy education, since the benefits of hybrid learning and co-creation [89] are well known. In response to growing promotions, self-learning has been implemented several times to evaluate models [24, 26, 27, 32, 46, 69, 82].

One study considered the colours of the plastic materials too bright, [45] another that the model was too fragile, [71] and two others pointed out the lack of anatomical variability when a single model was designed [25, 45]. Seven studies concluded that the anatomical detail was insufficient in their 3DPAM [28, 34, 45, 48, 62, 63, 81].

The segmentation and modelling time was considered very long and the cost very high (about 2000 USD) for more elaborate anatomical models of large and complex regions such as the retroperitoneum or the cervical region [27, 48]. In their study, Hojo and colleagues specified that it took 40 h to create their pelvic anatomical model [42]. The longest segmentation time was 380 h in the study by Weatherall and colleagues where several models were merged to make a finished paediatric airway model [36]. Segmentation and printing time was considered a drawback in nine studies [36, 42, 57, 58, 74]. However, 12 studies criticized the physical properties of their model, particularly its consistency, [28, 62] lack of transparency, [30] fragility and unicolor nature, [71] absence of soft tissues [66] or lack of detail [28, 34, 45, 48, 62, 63, 81]. These drawbacks could likely have been overcome with more segmentation or modelling time. Loss of acquisition-related information was an issue for three teams [30, 74, 77]. Patient data was used in which the iodinated contrast agent did not provide an optimal view of the blood vessels due to dose limitations [74]. The injected cadaveric model appears to be an ideal approach, freeing itself from the “as low as reasonably achievable” principle and limitations in the dose of contrast agent injected. 

### Limitations

Unfortunately, many articles did not mention certain key features of their 3DPAM. Less than half of the articles specified whether their 3DPAM was coloured or not. The printing scale was not consistently reported (43% of articles) and only 34% of articles mentioned the use of multiple materials. These printing parameters are crucial because they influence the 3DPAM’s pedagogical properties. Most of the articles did not provide enough information about the complexity of obtaining the 3DPAM (design time, qualifications of people, cost of software, cost of printing, etc.). This information is essential and must be taken into consideration before thinking about starting a project to develop a new 3DPAM.

## Conclusions

This systematic review demonstrates that the design and 3D printing of a normal anatomical model is feasible at a low cost, particularly by using FDM or SLA printers and inexpensive single-color plastic materials. These basic models can nevertheless be improved by adding colour, or adding structures made of various materials. More realistic models – printed with several materials of different colours and textures to reproduce the haptic qualities of the reference cadaveric model as closely as possible – require access to more expensive 3D printing technologies and substantially longer design time. This would greatly increase the overall cost. No matter the chosen printing process, selecting the appropriate imaging modality is key to successful 3DPAMs. The higher the spatial resolution, the more the model will match reality and be usable at advanced levels of study. From a pedagogical point of view, 3DPAMs are effective tools for teaching anatomy, as evidenced by knowledge tests carried out with students and by the students’ satisfaction. The pedagogical effectiveness of 3DPAMs seems to be best when they reproduce complex anatomical areas, and they are used by students early in their medical studies.

## Data Availability

The datasets generated and/or analysed for the current study are not publicly available due to the language barrier but are available from the corresponding author upon reasonable request.
